# The effects of fresh mango consumption on gut health and microbiome – Randomized controlled trial

**DOI:** 10.1002/fsn3.3243

**Published:** 2023-02-01

**Authors:** Pia Asuncion, Changqi Liu, Robert Castro, Viviana Yon, Martin Rosas, Shirin Hooshmand, Mark Kern, Mee Young Hong

**Affiliations:** ^1^ School of Exercise and Nutritional Sciences San Diego State University San Diego California USA

**Keywords:** fruit, gut health, gut permeability proteins, mango, microbiome, obese

## Abstract

Some individual fruits have been widely researched for their effects on overall health and correlations with chronic diseases. The beneficial effects of mango supplementation on metabolic diseases have been detected. However, research into mango consumption on gut health, including the microbiome, is limited to processed mango preparations or peels. Our goal was to examine the effects of fresh mango consumption on the gut microbiome, gut permeability proteins, and bowel movement habits in overweight/obese individuals. In a 12‐week crossover design study, 27 participants consumed 100 kcal/day of either mangos or low‐fat cookies with a washout period of 4 weeks. The mango intervention showed higher Shannon–Wiener and Simpson alpha diversity indices of the microbiome than the low‐fat cookie intervention in week 4. Significant differences in beta diversity of the microbiome were found between diet interventions at week 12. Mango consumption increased the abundance of *Prevotella maculosa*, *Corynebacterium pyruviciproducens*, and *Mogibacterium timidum* while it decreased *Prevotella copri*. Low‐fat cookie intake increased *Cyanobacterium aponinum* and *Desulfovibrio butyratiphilus* and reduced *Alloscardovia omnicolens*. There were no significant differences in circulating gut permeability protein (ZO‐1, claudin‐2, and occludin) levels. There was a slight increase in the amount of bowel movement with mango consumption, but no significant findings for frequency, consistency, strain, pain, and constipation in bowel movement between trials. Given these results, it can be concluded that consumption of mango may have positive effects on the gut health, which may yield possible health benefits for chronic disease that deserve further study.

## Graphical abstract

The objective of this study was to examine the effects of fresh mango consumption on the gut microbiome, gut permeability proteins, and bowel movement habits in overweight/obese individuals. In a 12‐week crossover design study, 27 participants consumed 100 kcal/day of either mangos or low‐fat cookies with a washout period of 4 weeks. This study suggests that consumption of mango may have positive effects on the gut health, which may yield possible health benefits for chronic disease that deserve further study.

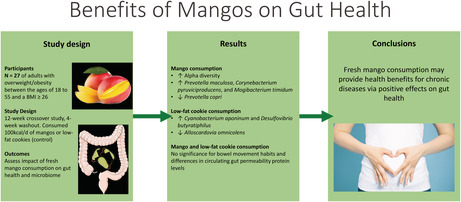

## 1 INTRODUCTION

The human gut microbiome has increasingly become a subject of research for its complexities and potential as a health indicator for various diseases. The gut microbiome has several functions within the body, including regulating the immune system and influencing chronic disease. For example, the gut microbiome regulates the homeostasis of cells in both the innate and adaptive immune systems (Wu & Wu, 2012). Additionally, diseases such as irritable bowel syndrome, inflammatory bowel disease, obesity, diabetes, cardiovascular disease, and cancer are heavily influenced by the gut microbiome (Hills et al., 2019). With many connections to health, the composition of the gut microbiome should be researched further.

Diet is an important influence on microbial composition and diversity. Partula et al. (2019) found that raw fruits and fish were associated with greater microbial diversity, while fried foods and sugary drinks were linked to lower microbial diversity. In an analysis of women migrating to the USA from Thailand, it was observed that immigrants had experienced a loss of gut microbiome diversity, function, and strain composition which resulted in a loss of fiber degradation capability, and a shift from *Prevotella* to *Bacteroide* dominance (Vangay et al., 2018). Studies have also shown that a lack of diversity, as well as abundance of certain bacterial species, is correlated with higher risk for gastrointestinal and chronic diseases (Hills et al., 2019). Individual food items such as fruit have been found to modify the abundance of certain phyla, genera, and species. A potential component of fruits that may be responsible for such changes is fiber, which provides substrates for microbial growth. Thus, fruits have the potential to enhance the diversity/abundance of the gut microbiome (Makki et al., 2018).

Mangos are rich in vitamins C and A, magnesium, potassium, bioactive phytochemicals (e.g., mangiferin, flavonoids, phenolic acids, and carotenoids), and fiber (Maldonado‐Celis et al., 2019; Ribeiro & Schieber, 2010). Mangos have been observed to promote many health benefits linked to inflammation and obesity, such as the reduction in pro‐inflammatory cytokines and C‐reactive protein (Fang et al., 2018; Rosas et al., 2022) and suppressing appetite while increasing satiety (Pinneo et al., 2022). The rich fiber content of mango may also exert a positive influence on the microbiome. For example, mango bars have been found to increase the abundance of certain microbial species, such as *Bifidobacterium*, *Prevotella*, and *Eubacterium* (Gutiérrez‐Sarmiento et al., 2020); and mango peels have yielded similar results with *Bifidobacterium* and *Lactobacillus* as well (Sáyago‐Ayerdi et al., 2019). However, there are no studies describing the prebiotic effects of fresh mango.

Obesity‐induced gut dysbiosis is associated with the impairment of tight junctions and increased gut permeability (Nagpal et al., 2018). Tight junction proteins play a pivotal role in the integrity of the gut barrier, which can affect immune function (Buckley & Turner, 2018; Lee et al., 2018). A lack of tight junctions can cause a defect in the integrity of the barrier increasing its permeability and allowing toxins to diffuse through, which leads to oxidative stress (Dokladny et al., 2016; Suzuki, 2020). Gut inflammation is associated with the downregulation of ZO‐1 and occludin, which may lead to a leaky gut (Bhat et al., 2019; Cereijido et al., 2007). Consumption of dietary components such as vitamin A, fiber, and polyphenols has demonstrated improvements in gut permeability (Khoshbin & Camilleri, 2020). These dietary components are present in mangos. However, there have been no studies on mango consumption and gut permeability in overweight/obese individuals.

Therefore, the objectives of this study were to examine the effects of fresh mango consumption as a whole fruit on the gut microbiome, gut permeability proteins, and bowel movement habits in overweight/obese individuals. It was hypothesized that consumption of 100 kcal/day of fresh mango for 12 weeks would significantly improve the gut microbiome, tight junction proteins, and bowel movements in comparison with more highly refined snack food. To our knowledge, this is the first study to assess the effects of fresh mango consumption on the gut microbiome and to explore its potential benefits for gut health.

## 2 MATERIALS AND METHODS

### 2.1 Participants

Participants were recruited using posted flyers in local public places. Details on participant information have been previously explained by Rosas et al. (2022). Briefly, there was a total of 27 participants (16 males and 11 females) with a mean age of 26.0 ± 8.1 years and a mean height of 172.4 ± 8.4 cm. The mango group had a mean body weight and BMI of 94.2 ± 14.7 kg and 31.6 ± 4.1 kg/m^2^, respectively; and the low‐fat cookie group had a mean body weight and BMI of 94.8 ± 14.5 kg and 31.9 ± 4.1 kg/m^2^, respectively. Inclusion criteria included being between the ages of 18–55 years and having a BMI of 26 kg/m^2^ or higher. Exclusion criteria were if the participants were smokers, pregnant, have metabolic or inflammatory diseases, or current use of antibiotics, probiotics, or prebiotics. This study was approved by the Institutional Review Board of San Diego State University and the trial was registered at clinicaltrials.gov [#NCT03957928].

### 2.2 Study design

**FIGURE 1:**
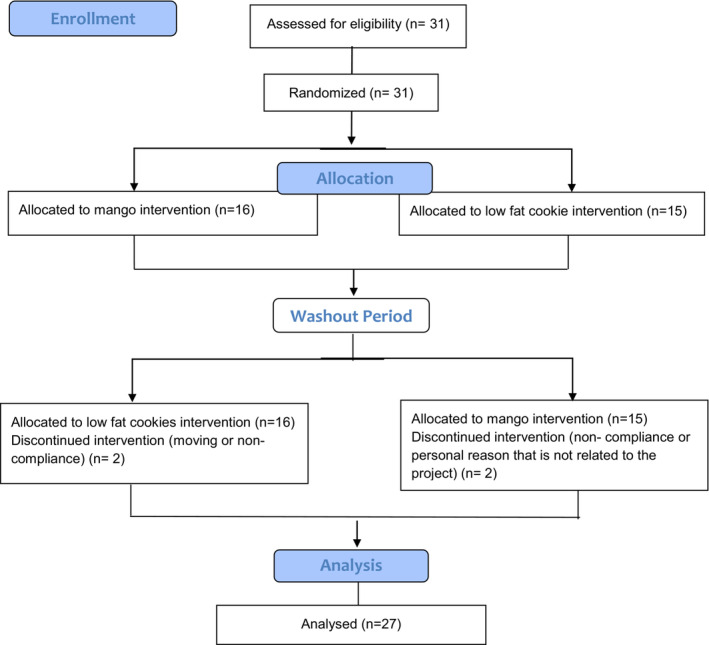
CONSORT flow diagram of participant selection.

This study followed a crossover design conducted over a 12‐week intervention with a washout period of at least 4 weeks (Figure 1). Participants were randomly assigned to either the mango group or the low‐fat cookie group. Participants consumed 100 kcal of fresh mangos consisting of 25 g carbohydrates (22.3 g sugar and 2.6 g dietary fiber), 1.35 g protein, and 0.6 g total fat, or 100 kcal portions of low‐fat Nilla wafer cookies (Nabisco) consisting of 20 g carbohydrates (10 g sugar and 0 g dietary fiber), 0.8 g protein, and 1.3 g total fat. The mangos and low‐fat cookies were provided as prepackaged samples. Participants were instructed to consume one pack per day for 12 weeks.

### 2.3 Stool collection

Stool samples were collected at home, stored under refrigeration, and transported within 24–48 h to our laboratory by the participants prior to visits at baseline, week 4, and week 12. Assembled stool preparation kits including gloves, sterile cotton swab sticks, and a stool container with DNA/RNA shield solution were provided to participants (Zymo Research). DNA isolation and quantification, and 16 rRNA gene sequencing were performed at Laragen Inc.

### 2.4 Gut permeability proteins

Participants were asked to fast 11–12 h prior to the collection of blood samples. Fasting blood samples were collected at baseline, week 4, and week 12 and centrifuged at 1200 *g* for 10 min at 4°C for serum separation. Serum samples were stored at −80°C. Human tight junction proteins, zonula occludens‐1 (ZO‐1), claudin‐2 (CLDN2), and occludin (OCLN) were measured using ELISA kits (MyBioSource).

### 2.5 Bowel movement questionnaire

Bowel movement habits were measured over seven consecutive days using a 5‐item questionnaire and a single question about the number of bowel movements the participant had each day. The five questions were as follows: “(1) How much stool did you produce?; (2) Please select the appropriate rating which best describes the consistency of the bowel movement; (3) Please select the appropriate rating which best describes your straining during this bowel movement; (4) please select the appropriate rating which best describes your pain during the bowel movement; (5) Please select the appropriate rating which best describes your overall feeling of constipation.” The rating scale of 1–7 corresponded to very soft to very hard (questions 2), none to extreme (questions 3 and 4), or not constipated to very constipated (question 5; Howarth et al., 2010).

### 2.6 Statistical analyses

The microbiome data were analyzed using R (version 4.1.0) and the Rstudio (version 1.4.1106) interface with the following packages: car, dplyr, edgeR, gglot2, ggpubr, igraph, MASS, phyloseq, phyloseqGraphTest, pscl, purr, readr, RioNorm2, tidyr, and vegan. Alpha diversity measures (Chao 1 richness index, ACE richness index, Shannon–Wiener diversity index, and Simpson diversity index) were analyzed using Friedman rank sum test followed by Wilcoxon signed rank exact test. Bray–Curtis index was used to assess beta diversity and analyzed using permutational multivariate analysis of variance (PERMANOVA). Principal coordinate analysis (PCoA) and graph‐based network analysis were used to visualize beta diversity. The differential abundance of taxa was analyzed using either a zero‐inflated Poisson model (for non‐over‐dispersed OTUs) or a zero‐inflated negative binomial model (for over‐dispersed OUTs). Data were filtered to remove taxa with <100 counts per million in over 90% of samples and were normalized based on relatively invariant OTUs prior to analysis (Ma et al., 2020). Differences were considered significant at *p* ≤ .05.

The gut permeability proteins and bowel movement data were analyzed using SPSS 27.0 (IBM). A two‐factor repeated measures ANOVA test was conducted to examine the effects of fresh mango or low‐fat cookie consumption on gut permeability proteins (ZO‐1, CLDN2, and OCLN) and bowel movement habits over time. Paired *t*‐tests were used to evaluate any difference in values between and within trials. If significant differences in baseline values were found using paired *t*‐test, ANOVA tests were performed to adjust the baseline for any identified covariates. Data were presented as mean ± SD (standard deviation) and *p* ≤ .05 was considered significant.

## 3 RESULTS

### 3.1 Alpha diversity

**FIGURE 2:**
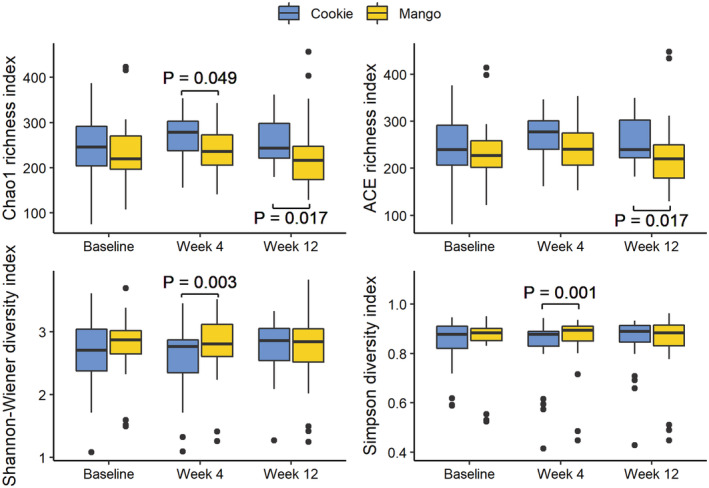
Alpha diversity measures of the microbiome. Box and the thick line therein denote quartiles and median. Whiskers extend 1.5 × interquartile range away from the first and third quartiles. Data beyond the whiskers are shown in dots.

The low‐fat cookie intervention showed higher Chao1 index at week 4 (*p* = .049) and week 12 (*p* = .017) and higher ACE index at week 12 (*p* = .017) as compared to the mango intervention (Figure 2). The mango intervention yielded greater Shannon–Wiener (*p* = .003) and Simpson (*p* = .001) indices at week 4.

### 3.2 Beta diversity

**FIGURE 3:**
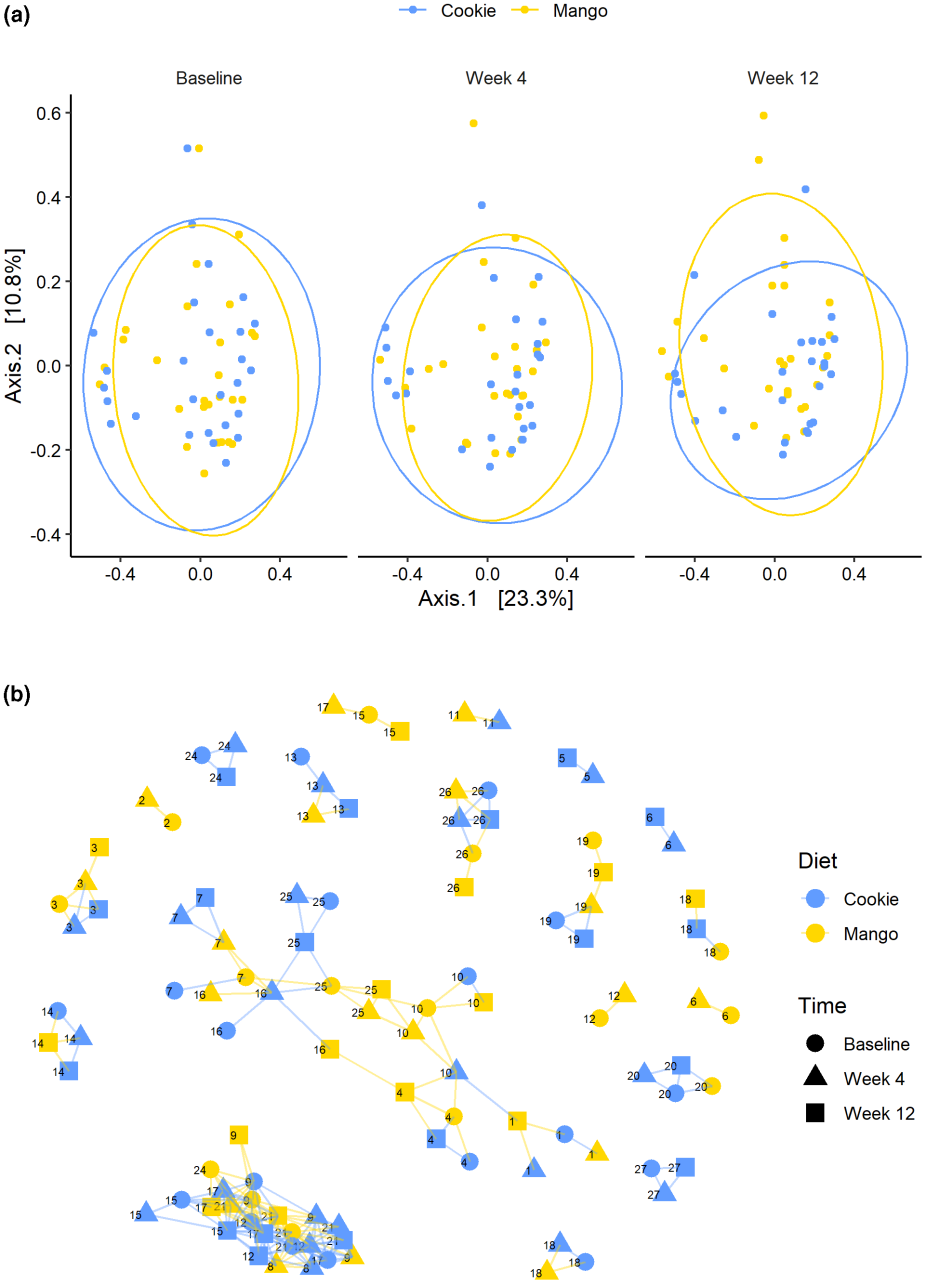
(a) Principal coordinate analysis of the Bray–Curtis index. Dissimilarities were significant at week 12 (*p* = .024). (b) A network was created by thresholding the Bray–Curtis matrix (maximum distance = 0.3).

Significant differences in the Bray–Curtis index were found between diet interventions (*p* = .005), but not between different time points or the interaction of the two. The significant difference in beta diversity between the mango and low‐fat cookie interventions was observed only at week 12 (*p* = .024). PCoA was performed to visualize the distance matrix. The first two principal coordinates (axis 1 and axis 2) of the PCoA explained 23.3% and 10.8% of variations of the data, respectively (Figure 3a). The PCoA plot also demonstrated that there was no clear separation between the diet interventions except in week 12 when samples in the mango intervention dispersed more along axis 2. We further analyzed the beta diversity index using graph‐based network analysis. As shown in Figure 3b, the samples mostly formed clusters by subjects instead of the interventions. This suggests that the greater diversity in microbiome composition between participants may diminish the treatment effects.

### 3.3 Differential abundance

**FIGURE 4:**
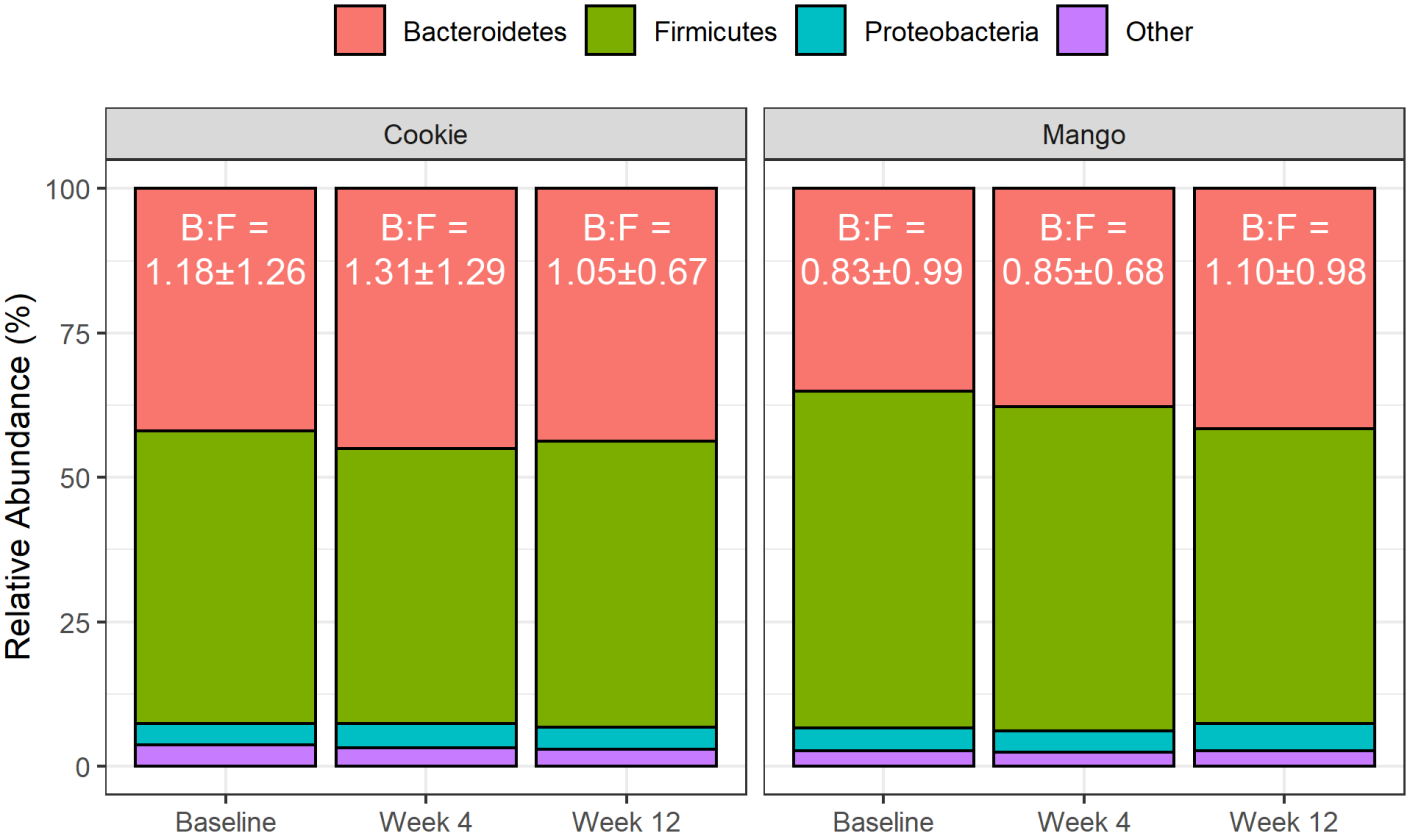
Relative abundance at phylum level and *Bacteroidetes*‐to‐*Firmicutes* (B:F) ratios.

Phylum‐level analysis indicates that the most abundant phyla in both the mango and low‐fat cookie interventions were *Firmicutes*, *Bacteroidetes*, and *Proteobacterium* (Figure 4). There were no significant differences in *Bacteroidetes*‐to‐*Firmicutes* ratio.

**FIGURE 5:**
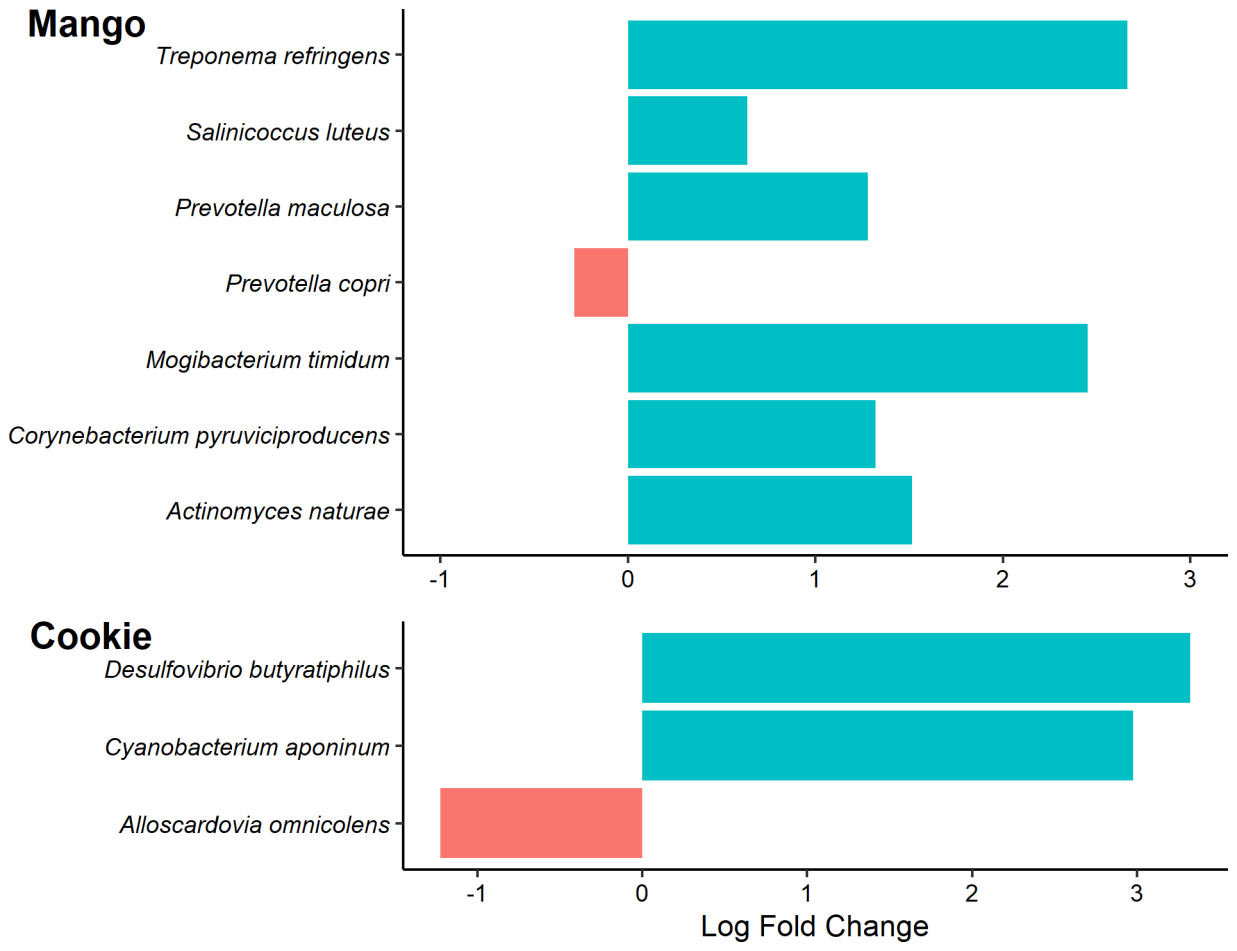
Taxa abundance changes between baseline and week 12 of the mango and low‐fat cookie interventions. All changes were significantly different at *p* ≤ .05.

Species‐level analysis of the top taxa changes is shown in Figure 5. Between baseline and week 12, the top species‐level taxa changes in the mango intervention led to a 1.5 log fold increase in *Actinomyces naturae* (*p* = .006), a 1.3 log fold increase in *Corynebacterium pyruviciproducens* (*p* = .042), a 2.5 log fold increase in *Mogibacterium timidum* (*p* = .042), a 0.3 log‐fold decrease in *Prevotella copri* (*p* = .042), a 1.3 log fold increase *Prevotella maculosa* (*p* = .001), a 0.6 log fold increase in *Salinicoccus luteus* (*p* = .014), and a 2.7 log fold increase in *Treponema refringens* (*p* = .003) between baseline and week 12. Between baseline and week 12, the top species‐level taxa changes in the low‐fat cookie intervention led to a 1.2 log fold decrease in *Alloscardovia omnicolens* (*p* = .017), a 3.0 log fold increase in *Cyanobacterium aponinum* (*p* = .002), and a 3.3 log fold increase in *Desulfovibrio butyratiphilus* (*p* = .017).

### 3.4 Permeability proteins

No significant differences in ZO‐1 and CLDN2 were observed between baseline and week 12 within or between the mango and low‐fat cookie interventions (Table 1). For OCLN, baseline adjusted analysis showed no significant differences between trials (Table 1).

**TABLE 1:** Effects of mango and low‐fat cookie consumption on permeability proteins (ZO‐1, CLDN2, and OCLN) and bowel movement habits.^†^

	Mango			Low‐fat cookie		
	Baseline	Week 4	Week 12	Baseline	Week 4	Week 12
Permeability proteins						
ZO‐1, ng/mL	2.54 ± 2.79	2.66 ± 4.25	3.05 ± 3.73	2.98 ± 5.66	3.20 ± 4.75	2.96 ± 4.52
CLDN2, ng/mL	1.39 ± 2.06	0.91 ± 1.00	1.16 ± 1.70	1.21 ± 1.57	0.96 ± 1.16	1.27 ± 2.46
OCLN, ng/mL	406 ± 236	473 ± 187	492 ± 203	472 ± 211	500 ± 248	426 ± 177
Bowel movement habits						
Frequency/day	1.69 ± 0.75	1.95 ± 0.86	1.78 ± 0.70	1.73 ± 0.64	1.73 ± 0.75	1.65 ± 0.68
Amount* (cups/day)	1.10 ± 0.60^a^	1.25 ± 0.73^b^	1.23 ± 0.88^b^	1.13 ± 0.49^a,b^	1.13 ± 0.6^a,b^	1.17 ± 0.72^a,b^
Consistency (Soft/Hard)	3.47 ± 0.78	3.50 ± 0.75	3.49 ± 1.00	3.45 ± 0.86	3.44 ± 0.92	3.43 ± 0.85
Strain	2.23 ± 0.87	2.25 ± 1.04	2.11 ± 1.00	2.46 ± 0.94	2.26 ± 1.05	2.21 ± 0.86
Pain	1.51 ± 0.80	1.73 ± 1.10	1.53 ± 0.91	1.60 ± 1.03	1.70 ± 1.02	1.65 ± 0.87
Constipation	1.86 ± 0.96	2.06 ± 1.02	1.96 ± 1.14	2.11 ± 0.87	2.07 ± 1.18	1.90 ± 0.84

Abbreviations: CLDN2, claudin‐2; OCLN, occludin; ZO‐1, zonula occludens‐1.

^†^ Values are expressed as mean ± SD.

* Superscripts of different letters denote significance at *p* ≤ .05.

### 3.5 Bowel movement habits

There were no significant differences in the frequencies of bowel movements in the mango or low‐fat cookie interventions (Table 1). There was a slight increase in the estimated amount of stool produced from baseline to week 4 and week 12 (*p* < .05) in the mango intervention. There were no significant differences in the amount of stool produced from baseline to week 12 in the low‐fat cookie intervention. There were no significant differences found within either the mango or low‐fat cookie trials in bowel movement regarding consistency (soft or hard), strain, pain, or constipation. Similarly, there were no significant differences found in the comparison between mango and low‐fat cookie interventions.

## 4 DISCUSSION

This study examined the effects of daily mango consumption for 12 weeks on the gut microbiome, gut permeability proteins, and bowel movement habits compared to control, low‐fat cookie consumption.

### 4.1 Alpha diversity

The mango intervention decreased the richness‐based alpha diversity measures (Chao1 and ACE indices) while increasing the species evenness (Shannon–Wiener and Simpson indices) in comparison with the low‐fat cookie intervention. This may indicate that although the mango intervention resulted in fewer members of species as compared to the low‐fat cookie intervention, it led to a more even distribution of abundance across the species. Since species richness and evenness are both important determinants of microbial diversity, the Shannon–Wiener and Simpson indices provide more inference about the community composition (Kim et al., 2017). The increased Shannon–Wiener and Simpson indices were observed within the first 4 weeks of mango intervention.

Related literature shows conflicting results regarding microbial richness to that of the present study. An intervention of 400 mL of fruit and vegetable juice per day for 3 weeks showed a significant increase in alpha diversity using the Chao1 metric (Choi et al., 2018). A correlation study also showed raw fruit consumption to increase richness using the same index (Partula et al., 2019). This may be, in part, due to other factors affecting the gut microbiome since the dietary interventions were added to participants' regular diet. Despite the conflicting results in the Chao1 index for the mango intervention, other literature does support the week 4 results of our study. Using the Simpson index for richness and evenness, Partula et al. (2019) revealed positive associations between raw fruit consumption and alpha diversity. Likewise, fresh fruits such as berries and grapes, citrus fruits, and stone fruits were linked to an increased alpha diversity using the Shannon–Wiener index (van Soest et al., 2020). Since mangos are stone fruits, the significant increase at week 4 in alpha diversity coincides with the related literature.

In the Shannon–Wiener and Simpson indices, the alpha diversity was significantly higher at week 4 of the mango intervention compared to the low‐fat cookie intervention. Findings from Hills et al. (2019) indicate that a lack of microbial diversity is connected to increased systemic inflammation, cardiovascular and diabetic symptoms, and irritable bowel syndrome. Since no difference between the interventions was detected in week 12 and since the diversity did not change over time within an intervention, care should be taken in over‐interpreting the results.

### 4.2 Beta diversity

In addition to examining the richness and evenness of the microbiome, dissimilarities in microbial diversity between subjects were analyzed using the Bray–Curtis index. A significant difference in beta diversity between diet interventions was found at week 12, meaning that the dissimilarity of diversity between the low‐fat cookie and the mango interventions was greatest at that time point. In a study evaluating the diet of healthy French adults, beta diversity dissimilarities were influenced by fruit consumption (Partula et al., 2019). Beta diversity indicated significantly greater differences in Egyptian diets that included more fruits and vegetables than in Western diets (Shankar et al., 2017). Significant dissimilarities found at week 12 between diet interventions may correspond with these studies due to the dissimilarities being driven mainly by fruits and vegetables.

### 4.3 Relative abundance

The four phyla that make up most of the gut bacteria are *Proteobacteria*, *Actinobacteria*, *Bacteroidetes*, and *Firmicutes*, with the latter two making up more than 90% of the bacteria found within the gut (Rinninella et al., 2019). Metagenomic data suggest that 25% of microbial genes associated with obesity belong to *Firmicutes*, whereas 42% of the genes associated with leanness belong to *Bacteroidetes* (Krajmalnik‐Brown et al., 2012). The *Bacteroidetes*‐to‐*Firmicutes* ratio (B:F) has been used as a health indicator, with a greater relative abundance of *Firmicutes* in comparison with *Bacteroidetes* being associated with higher risks of obesity and cardiovascular disease (Magne et al., 2020). Neither the low‐fat cookie nor mango intervention showed a significant change in B:F ratio.

At the species level, *P. maculosa* increased with the mango intervention. Chen et al. (2021) suggested beneficial roles of *P. maculosa* in glucose metabolism, and Liu et al. (2020) suggested that *P. maculosa* plays an important role in immune function. Another study also reported favored growth of the *Prevotella* genus after consuming 100 g of dehydrated mango bars that included both pulp and peel of the Ataulfo mango (Gutiérrez‐Sarmiento et al., 2020). However, the *Prevotella* genus has high genetic diversity, with several members being pathobionts that promote several diseases (Precup & Vodnar, 2019). For example, the expansion of intestinal *P. copri* has been associated with the pathogenesis of arthritis (Pianta et al., 2017; Scher et al., 2013). In the present study, the prevalence of *P. copri* was decreased after 12 weeks of the mango intervention. Additionally, the mango treatment increased the abundance of *M. timidum*, which is abundant in healthy rats compared to rats with ulcerative colitis (El‐Baz et al., 2020), and *C. pyruviciproducens*, which has been suggested to be an immune modulator as it promotes macrophage activity and upregulates antibody responses (Tong et al., 2012). Health implications of the other species that increased following the mango intervention remain unclear. Similarly, little is known about the taxa changes in the low‐fat cookie intervention.

### 4.4 Gut permeability proteins

Tight junction proteins play a critical role in the structure of the intestinal lining. Obesity is a factor influencing the abundance of tight junctions and may account for the prevalence of leaky gut and intestinal hyperpermeability (Ahmad et al., 2017; Mujawdiya et al., 2020). In a cohort of 122 severely obese and nonobese individuals, it was found that obese individuals had tight junction impairment in the small intestine due to a lack of occludin and tricellulin (Genser et al., 2018). Moreover, Mujawdiya et al. (2020) found that mango seed kernel extract reversed intestinal hyperpermeability, as measured by ZO‐1 and claudin‐1, and improved obesity‐related metabolic symptoms, whereas a high‐fat diet reduced the expression of tight junctions, and consequently led to metabolic disease in obese mice. Although no significance was found in circulating gut permeability proteins in the present study, this is the first study examining mango fruit consumption on tight junction proteins. Our population was relatively healthy (i.e., free of metabolic and inflammatory disease) except for being overweight/obese, resulting in nonsignificant outcomes. Future directions include the effects of mango consumption on microbiome and gut permeability in patients with gut inflammation and the relationship between them.

### 4.5 Bowel movement habits

Frequency and consistency did not change throughout the intervention, nor were there any differences between the two interventions. However, the mango group did have a slightly increased amount of stool produced from baseline, whereas the low‐fat cookie intervention did not. Mangos contain fiber, which is known to have a bulking action that is likely responsible for the increased volume of stool seen in the mango group (de Lourdes García‐Magaña et al., 2013; Yang et al., 2012). In a meta‐analysis by Yang et al. (2012), it was shown that increasing dietary fiber intake can also improve bowel movement frequency in individuals with constipation. The participants in the present study were not constipated, nor did they have any strain or pain associated with their bowel movements. Future directions may include evaluating the effects of mango consumption on bowel movement habits in individuals suffering from constipation.

## 5 CONCLUSIONS

This study found that daily consumption of 100 g fresh mango increased the diversity of the gut microbiome after 4 weeks, with the greatest dissimilarities between diet interventions at week 12. Mango consumption results in an increased abundance of *P. maculosa*, *C. pyruviciproducens*, and *M. timidum* and a reduction in *P. copri*, while low‐fat cookie intake increased *C. aponinum* and *D. butyratiphilus* and reduced *A. omnicolens*. These results indicate fresh mango consumption may elicit positive benefits for gut health, which may have positive implications for chronic diseases such as systemic inflammation, cardiovascular diseases, diabetes, and irritable bowel syndrome. While this study provides valuable insight into the benefits of fresh mango consumption, it is not without its limitations. This study had a low sample size, and it did not include participants with a BMI <26. Future studies should aim for a larger sample size and include participants with a BMI <26. Additionally, future studies may consider evaluating the effects of fresh mango consumption at varying amounts (e.g., 50, 100, and 150 g), and compare the effects of whole mango with other fruits.

## CONFLICT OF INTEREST STATEMENT

The authors declare that they do not have any conflict of interest.

## ETHICAL APPROVAL

This study was approved by the Institutional Review Board of San Diego State University. Written informed consent was obtained from all study participants.

## Data Availability

The data that support the findings of this study are available from the corresponding author upon reasonable request.
